# Explosive Output to Enhance Jumping Ability: A Variable Reduction Ratio Design Paradigm for Humanoid Robot Knee Joint

**DOI:** 10.3390/biomimetics11010045

**Published:** 2026-01-06

**Authors:** Xiaoshuai Ma, Qingqing Li, Haochen Xu, Xuechao Chen, Junyao Gao, Fei Meng

**Affiliations:** 1 School of Mechatronic Engineering, Beijing Institute of Technology, Beijing 100081, China; 3220215026@bit.edu.cn (X.M.); bhr_liqingqing@bit.edu.cn (Q.L.); 3120235413@bit.edu.cn (H.X.); chenxuechao@bit.edu.cn (X.C.); gaojunyao@bit.edu.cn (J.G.); 2 Key Laboratory of Biomimetic Robots and Systems, Ministry of Education, Beijing 100081, China

**Keywords:** humanoid robot, explosive jumping, knee joint design, linear actuator

## Abstract

Enhancing the explosive power output of the knee joints is critical for improving the agility and obstacle crossing of humanoid robots. However, a mismatch between the knee-to-CoM transmission ratio and jumping demands, together with power-loss–induced motor performance degradation at high speeds, shortens the high-power operating window and limits jump performance. To address this, this paper introduces a variable-reduction-ratio knee-joint paradigm in which the reduction ratio is coupled to the joint angle and decreases during extension. Analysis of motor output and knee kinematics motivates coupling the reduction ratio to the joint angle. A high initial ratio increases the takeoff torque, and a gradual decrease limits motor speed and power losses, extending the high-power window. A linear-actuator-driven guide-rod mechanism realizes this strategy, and parameter optimization guided by explosive jump control is employed to select the design parameters. Experimental validation demonstrates a high jump of 0.63 m on a single-joint platform (a theoretical improvement of 31.9% over the optimal fixed-ratio baseline under the tested conditions). Integrated into a humanoid robot, the proposed design enables a 1.1 m long jump, a 0.5 m high jump, and a 0.5 m box jump.

## 1. Introduction

Jumping is essential to humanoid locomotion yet remains difficult. It enables the rapid traversal of complex terrains, overcoming large obstacles, and improving mobility [1,2,3]. Recent advances in actuation and control have improved performance [4,5], yet humanoid robots still fall short of human-level jumping.

The hydraulically actuated Boston Dynamics Atlas currently demonstrates among the most advanced jumping capabilities, achieving vertical jumps over 0.6 m and agile parkour [6]. However, hydraulic systems face leakage, noise, cost, and mass issues. Reports also indicate a shift away from the hydraulic version [7].

As the current mainstream approach, electrically driven humanoid robots, which are primarily actuated by permanent magnet synchronous motors (PMSMs), offer advantages such as high efficiency, precise control, low noise, and lightweight design [8]. However, as shown in Table 1, their jumping capabilities remain considerably lower than humans.

During jumping, sufficient kinetic energy must be generated within 0.15–0.50 s [9], imposing high demands on leg joints, especially the knee [10,11,12,13]. High-power motors are large and heavy, increasing knee inertia and reducing responsiveness. Designing a compact, lightweight knee joint that still delivers explosive power remains challenging.

In electrically actuated humanoids, the knee joint is typically driven by a rotary or a linear actuator. Rotary designs pair a motor with a planetary, harmonic, or cycloidal drive. Planetary gearboxes are compact and efficient [14,15], but added mass increases leg inertia. To boost knee torque without adding distal inertia, platforms such as Unitree H1 [16] and the electric Atlas [17] mount high-torque motors near the hip and transmit power through linkages. Unitree H1 reports 350 Nm peak knee torque and can perform backflips [18]. Other platforms, including Unitree G1 [19], Engine AI [20], and the MIT humanoid [21], reduce size and mass to lower knee loading and improve agility, enabling martial arts [22], flips [18], and a 0.5 m vertical jump [21]. The cable-driven JAXON-3P [23] uses a harmonic drive and tendons to halve leg mass and reach 700 Nm peak torque but achieves only a 0.3 m vertical jump. At the World Robot Competition in China [24], RobotEra L7 [25] used oversized motors and a proximally located knee actuator; with increased hip contribution and a split-leg takeoff, it achieved a 0.95 m vertical jump. Its long jump attempt reached 1.40 m but was annulled for a rules violation. MagicAtom Z1 achieved a 0.52 m vertical jump by leg tucking [26]. Noetix Robotics N2 achieved a 1.25 m long jump [27]. Although these designs enhance dynamics, their motor power utilization remains low.

**Table 1:** Specifications and jumping performance of electrically actuated humanoids with high jumping capability.

Robot	Mass/Height (kg/m)	Knee Peak Torque (Nm)	Vertical/Long/Box (m) Jump
Unitree H1 [16]	47/1.8	360	0.3 */–/–
Unitree G1 [19]	35/1.3	120	0.3 */1.4/–
MIT-Humanoid [21]	24/1.04	144	0.5/–/–
JAXON 3-P [23]	35/1.7	700	0.3/–/–
Noetix Robotics N2 [27]	30/1.2	200	–/1.25/–
MagicBot Z1 [26]	40/1.4	130	0.52/–/–
RobotEra L7 [25]	65/1.71	400	0.95/1.4/–
Cassie [28]	27.2/1.3	–	0.5/1.4/0.4
BHR8-J1 (ours)	45/1.6	318	0.5/1.1/0.5

* Estimated values based on published specifications. – No publicly available data. Jump tasks: Vertical denotes vertical high jump, Long denotes standing long jump, and Box denotes box jump onto a fixed-height platform.

In typical human-like leg kinematics, the transmission ratio from the knee joint to the center of mass (CoM) increases with joint extension, which conflicts with the jumping requirement for high torque followed by high speed [3]. Furthermore, motor torque is limited at low speeds and power output is insufficient at high speeds, exacerbating this mismatch. Since rotary actuator joints have fixed or only slightly varying reduction ratios, they cannot address this issue. As a result, the knee motor’s performance cannot be fully exploited during jumping, leading to poor jumping capability.

In addition, Cassie adopts parallel rotary vector reducers and integrates elastic elements to reduce motor speed demands, enabling vertical jumps of 0.5 m, forward jumps of 1.4 m, and box jumps of 0.4 m [28]. Nevertheless, its large leg volume limits terrain adaptability, and adding an upper body significantly increases weight, degrading overall jumping performance [29].

Linear actuators typically integrate PMSMs with roller screws or ball screws. Robots such as Optimus [30], Lola [31], and RH5 [32] utilize linear actuators and demonstrate stable walking. However, their designs primarily focus on torque output rather than dynamic motion, limiting their capability for explosive jumping.

Small-scale robots improve jumping via parallel springs [33], parallel mechanisms [34], and momentum-boosting [35]. These methods face scale-up challenges for full-sized humanoids due to structural complexity, long transmission chains, limited elastic energy density, and control difficulty.

This paper presents a design paradigm for an electrically driven humanoid knee joint aimed at enhancing jumping ability. By analyzing the knee’s kinematic characteristics and the motor’s output capability during the takeoff phase, we establish a coupling between the joint angle and reduction ratio to boost motor output during takeoff and thus improve jumping performance. Based on this concept, we propose a practical structural implementation and a parameter optimization method guided by jump control strategies. The effectiveness of the approach is validated through numerical simulations, experiments on a single-leg test platform, and trials with a full-scale humanoid robot.

Unlike most existing variable-transmission and linkage-based leg designs that start from a fixed mechanism and then optimize its parameters, our approach first identifies, through analysis of the knee–CoM transmission ratio and the PMSM torque–power envelope, the desired qualitative profile of the reduction ratio for explosive jumping: a high ratio at the beginning of extension followed by a monotonic decrease. From a biomechanical perspective, this jump-specific profile is consistent with the observation that human knee moment arms and joint power during jumping tend to peak around mid-extension. The proposed linear-actuator-driven guide-rod mechanism is then chosen because it can realize this profile with high parametric tunability through a small set of geometric variables.

The major contributions are as follows:

- An explosive variable reduction ratio knee joint (EVRR-KJ) strategy in which the reduction decreases with extension, sustaining high knee motor power throughout jumping.
- A compact linear-actuator-driven knee mechanism with simple structure and high parametric flexibility that realizes diverse reduction-ratio curves.
- A parameter optimization method guided by explosive jump control strategies to identify optimal reduction-ratio curves for improved jumping under the same motor and control assumptions as an optimized fixed-ratio baseline.

The remainder is organized as follows. Section 2 presents the EVRR-KJ strategy. Section 3 proposes the structural implementation. Section 4 introduces the parameter optimization. Section 5 describes experiments. Section 6 concludes and outlines future work.

## 2. EVRR-KJ Strategy for Explosive Jumps

To improve knee output during takeoff, we analyze motor output characteristics and knee kinematics, and propose a heuristic strategy that couples the reduction ratio to the joint angle.

### 2.1. Motor Output Characteristics Analysis

PMSMs are widely used as primary actuators in legged robots, and their peak output is mainly limited by power losses and thermal constraints. The total motor power loss comprises copper, iron, and mechanical losses:

(1) $$P_{\mathrm{loss}}=P_{\mathrm{copper}}+P_{\mathrm{iron}}+P_{\mathrm{mechanical}}.$$

Here, $P_{\mathrm{copper}}$ denotes the winding resistance loss (current-related), $P_{\mathrm{iron}}$ represents hysteresis and eddy-current losses (speed-related), and $P_{\mathrm{mechanical}}$ refers to friction and windage losses (also speed-related).

**Figure 1:**
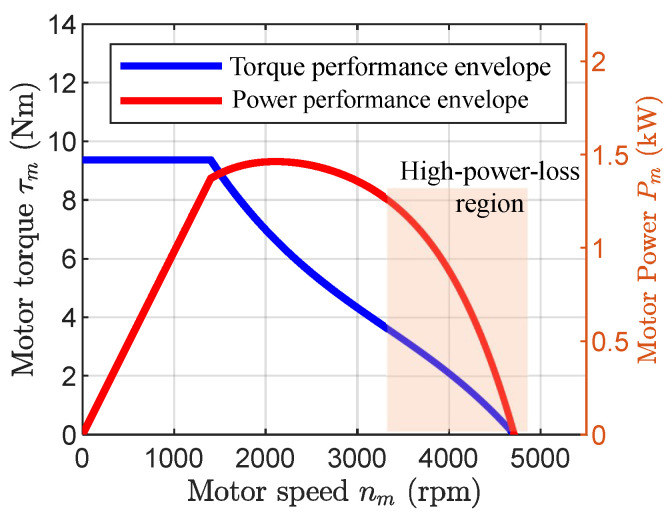
Motor torque performance envelope (TPE) and power performance envelope (PPE), with the high-loss region highlighted.

As illustrated in Figure 1, at low speeds, the peak output is thermally limited by the maximum allowable current, yielding an approximately constant peak torque. In the mid-speed region, the output is constrained by the rated power, resulting in an approximately constant peak power. At high speeds losses rise rapidly and output power drops steeply. To maximize output during jumping, operating near the mid-speed region avoids thermal and excessive-loss constraints. In this paper, these low-, mid-, and high-speed regions are defined empirically from the manufacturer-provided torque–speed–power envelope: the low-speed region corresponds to the current-limited constant-torque area, the mid-speed region corresponds to the rated constant-power area, and the high-speed region corresponds to motor speeds beyond the constant-power area where iron and mechanical losses cause a rapid drop in available power.

The relation between joint output power and motor power is

(2) $$P_{J}=\eta_{J}P_{m},$$

where $P_{J}$ is the joint output power, $P_{m}$ is the motor power, and $\eta_{J}$ is the transmission efficiency determined by the reduction mechanism.

### 2.2. Kinematic Analysis of the Knee Joint During Jumping Based on a Simplified Model

**Figure 2:**
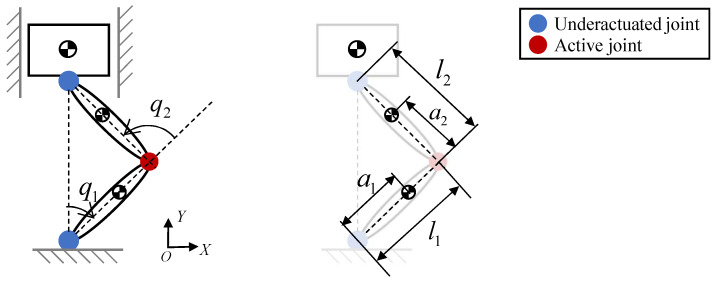
Prototype system used to analyze knee joint requirements in jumping.

A simplified leg model is used to capture the main motion characteristics. As shown in Figure 2, the knee is modeled as the only actuated joint, while the hip and ankle are passive and constrained to vertical motion. This simplification enables focused analysis of the knee contribution to the propulsion of the center of mass (CoM) during takeoff.

Takeoff is along the *y*-axis. The CoM velocity in this direction is

(3) $${\dot{y}}_{\mathrm{CoM}}=J_{\mathrm{CoM},y}\left(q_{2}\right)\;{\dot{q}}_{2},\;J_{\mathrm{CoM},y}\left(q_{2}\right)=\frac{\partial \;y_{\mathrm{CoM}}\left(q_{2}\right)}{\partial q_{2}}\;.$$

Here, ${\dot{y}}_{\mathrm{CoM}}$ is the CoM velocity in the *y* direction, $J_{\mathrm{CoM},y}\left(q_{2}\right)$ is the Jacobian component with respect to the knee joint angle $q_{2}$, and ${\dot{q}}_{2}$ is the knee angular velocity.

Therefore, the transmission ratio from the knee to the CoM along the *y*-axis is

(4) $$\lambda \left(q_{2}\right)=\frac{{\dot{q}}_{2}}{{\dot{y}}_{\mathrm{CoM}}}=\frac{1}{J_{\mathrm{CoM},y}\left(q_{2}\right)}\;.$$

The Jacobian is

(5) $$J_{\mathrm{CoM},y}\left(q_{2}\right)=\frac{\left[a_{1}m_{1}+\left(l_{1}+a_{2}\right)m_{2}+\left(l_{1}+l_{2}\right)m_{3}\right]\sin\left(q_{2}/2\right)}{m_{1}+m_{2}+m_{3}}\;,$$

where $a_{1}$ and $a_{2}$ are the distances from the shank and thigh CoM to the previous joint, $l_{1}$ and $l_{2}$ are the shank and thigh lengths, and $m_{1}$, $m_{2}$, $m_{3}$ are the masses of the shank, thigh, and torso, respectively.

**Figure 3:**
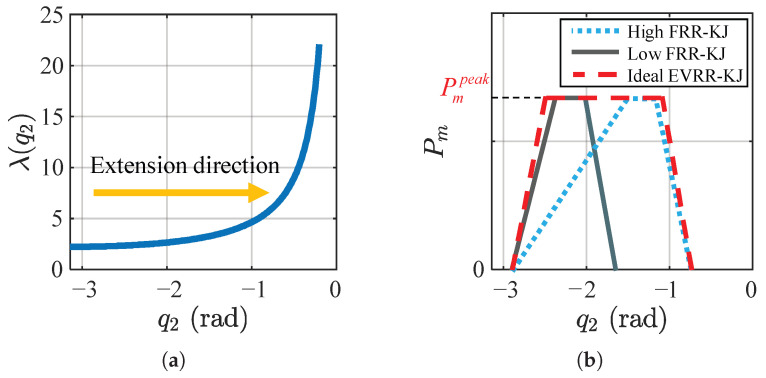
(**a**) Knee–CoM *y*-axis transmission ratio $\lambda (q_{2})$ vs. knee angle $q_{2}$; (**b**) mechanism underlying the high-burst output of EVRR-KJ.

From Equations (4) and (5), the transmission ratio $\lambda (q_{2})$ increases with $q_{2}$ (Figure 3a), and its rate of increase also grows with $q_{2}$. Once $q_{2}$ exceeds −1rad, $\lambda (q_{2})$ rises sharply. Because the numerator of $J_{\mathrm{CoM},y}\left(q_{2}\right)$ in Equation (5) depends linearly on the link lengths and masses, reasonable uncertainties in limb lengths and mass distribution primarily scale the magnitude of $\lambda (q_{2})$ but do not change its monotonic increase with knee extension or the sharp rise once $q_{2}$ exceeds about −1rad. For this reason, the qualitative conclusions drawn from $\lambda (q_{2})$ are robust to such parameter variations.

The CoM force and velocity along the *y*-axis are

(6) $$F_{\mathrm{CoM},y}=\tau_{2}\;\lambda \left(q_{2}\right),\;{\dot{y}}_{\mathrm{CoM}}={\dot{q}}_{2}/\lambda \left(q_{2}\right),$$

where $\tau_{2}$ is the knee torque.

A fixed-reduction-ratio knee joint (FRR-KJ) presents a trade-off: a low ratio yields insufficient takeoff acceleration and delays entry into the constant-power region, whereas a high ratio requires impractically high motor speed late in the jump and fails to sustain high power (Figure 3b). To address this, we propose an EVRR-KJ strategy with a ratio that progressively decreases during extension. This enables rapid entry into, and maintenance within, the high-power (constant-power) region at takeoff, improving jump performance (Figure 3b).

As in Equation (5), increasing CoM height and limb lengths raises $J_{\mathrm{CoM},y}\left(q_{2}\right)$, which lowers $\lambda (q_{2})$ and the required motor speed, thereby reducing apparent leg inertia and improving dynamics [14]. These parameters should match system-level design requirements.From a biomechanical perspective, human knee moment arms and joint power during jumping tend to peak around mid-extension, which motivates a reduction ratio that is high at the beginning of extension but decreases thereafter, so that the electric motor can emulate the human knee’s ability to deliver high power over a relatively narrow angular window.

It should be emphasized that this simplified 1-DoF model is not intended as a high-fidelity predictor of absolute jump height, but rather as an analytical tool to reveal how the knee–CoM transmission ratio $\lambda (q_{2})$ evolves with knee angle and how this interacts with the motor torque–power envelope. The full-scale robot experiments later in this paper serve to validate that the design paradigm derived from this simplified analysis can be realized in hardware.

## 3. Mechanical Design

**Figure 4:**
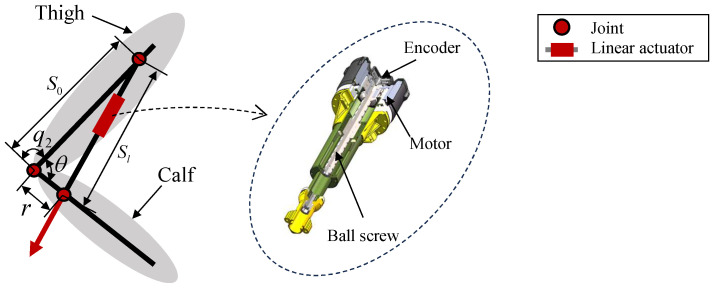
Schematic of the EVRR-KJ with a high-efficiency linear actuator.

To realize the above strategy, we use a linear-actuator-driven guide-rod mechanism to generate knee rotation, as shown in Figure 4. The linear actuator (right subfigure of Figure 4) comprises a frameless torque motor, a high-efficiency dual-lead ball-screw system [36], and an encoder. The ball nut is rigidly coupled to the motor rotor; rotor rotation drives the nut, while the screw translates axially to produce linear output. To improve compactness, a pair of duplex angular-contact bearings both supports the rotor’s rotation and provides sufficient axial stiffness for the linear output. The reduction ratio as a function of the knee angle is

(7) $$k=\frac{2\pi rS_{0}\sin\theta }{Q\sqrt{S_{0}^{2}+r^{2}-2S_{0}r\cos\theta }}.$$

Here, *Q* is the ball-screw lead, *r* is the crank length, θ is the angle between the crank and the frame, and $S_{0}$ is the frame length. Equation (7) follows from the geometry of the guide-rod and linear-actuator mechanism in Figure 4 by writing the loop-closure equation for the linkage, expressing the screw displacement as a function of θ, and differentiating to relate the motor-side and joint-side velocities. For the parameter ranges and knee-joint angles considered in this study, θ remains within an interval that avoids the singular configurations at which the denominator of Equation (7) would approach zero, so the mechanism does not encounter kinematic singularities in either simulation or experiment. The relationship between the knee joint angle $q_{2}$ and θ is

(8) $$q_{2}=\theta -\pi .$$

**Figure 5:**
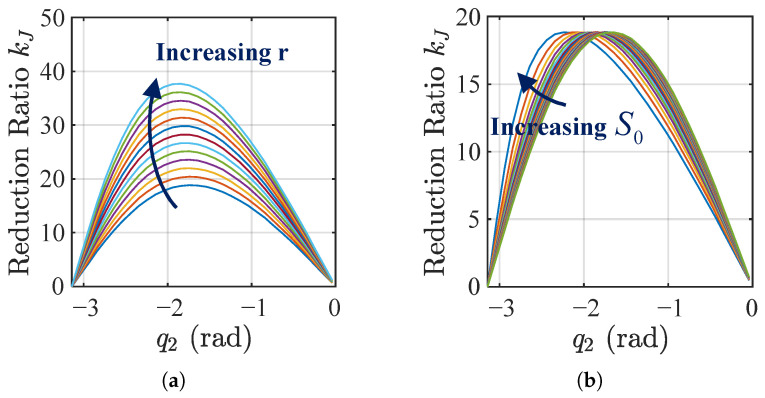
Effect of structural parameters on the coupling between joint angle and reduction ratio (*Q* = 10 mm). (**a**) Effect of *r* with $S_{0}$ = 250 mm; *r* varies from 10 to 50 mm. (**b**) Effect of $S_{0}$ with *r* = 30 mm; $S_{0}$ varies from 150 to 250 mm.

According to Equation (7), Figure 5 illustrates how design parameters affect the coupling between reduction ratio and joint angle. For the parameter ranges considered, the reduction ratio generally increases and then decreases as the joint extends. Varying the link length *r* changes the peak reduction ratio (Figure 5a), while the minimum approaches zero near singular configurations. Adjusting the offset $S_{0}$ shifts the joint angle at which the maximum reduction ratio occurs within [−π,−π/2] (Figure 5b). These results demonstrate wide parameter tunability, enabling a decreasing reduction ratio in extended poses that meets the kinematic requirements of this study.

Additionally, the coupling can be further tuned by adjusting the angle between the hinge line and the joint axis, increasing design flexibility. In this case,

(9) $$q_{2}=\theta -\pi +\Delta \theta ,$$

where Δθ denotes the angular offset introduced during initial assembly.

## 4. Joint Parameter Optimization Based on Explosive Jumping Control

In this section, we optimize the knee-joint structure using a parameter optimization framework based on the simplified model and compare it with an optimal fixed-ratio scheme.

### 4.1. Optimization Method

**Figure 6:**
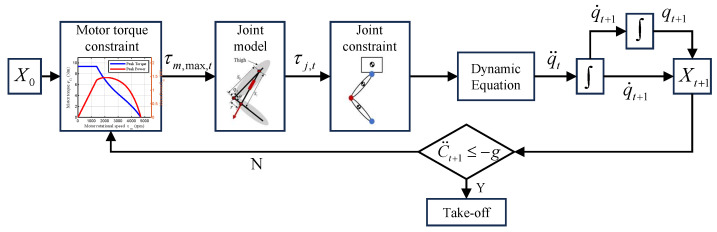
Explosive jump control process for parameter optimization.

To maximize motor output during takeoff, we implement an explosive control strategy [12] that drives the joint at its maximum available torque, as illustrated in Figure 6. Based on the output characteristics of permanent-magnet synchronous motors (PMSMs), the motor torque is bounded by current, power, thermal, and speed/voltage limits:

(10) $$\tau_{m,\max}=\left\{\begin{array}{cc} \tau_{\mathrm{peak}}, & \omega_{m}\leq \omega_{\mathrm{break}},\\ \tau_{\mathrm{limit}}\left(I_{q},\omega_{m}\right), & \omega_{m}\in \left(\omega_{\mathrm{break}},\omega_{\max}\right], \end{array}\right.$$

where $\tau_{m}$ is the motor torque, $\tau_{\mathrm{peak}}$ is the peak torque, $\omega_{m}$ is the motor angular velocity, and $I_{q}$ is the *q*-axis current. Here, $\omega_{\mathrm{break}}$ denotes the speed at which the motor enters the power-limited region, and $\omega_{\max}$ is the theoretical maximum speed. The function $\tau_{\mathrm{limit}}\left(I_{q},\omega_{m}\right)$ gives the maximum torque deliverable in the power-limited region. The maximum-torque strategy is deliberately chosen as a conservative design case: it represents an extreme scenario in which the knee joint is most prone to driving the motor into the high-speed, high-loss region. If the joint can sustain high power and avoid excessive motor speeds under this strategy, less aggressive and more sophisticated controllers for whole-body coordination, which generally require lower peak joint speeds for the same jump performance, will also operate the actuator within a favorable region of its torque–speed envelope. The overall parameter optimization procedure is summarized in Algorithm 1.

Given the high efficiency of the ball-screw transmission, we neglected drivetrain losses in the optimization and modeled the joint torque as

(11) $$\tau_{J}=\tau_{m}\;k_{J}\left(q_{2}\right).$$

Modern ball-screw and precision gearbox drives typically exhibit efficiencies on the order of 90% or higher, so a first-order neglect mainly affects absolute values rather than relative comparisons between FRR-KJ and EVRR-KJ [36]. Moreover, drivetrain losses grow with motor speed, so accounting for them would further penalize high-speed operation and thus make a decreasing reduction ratio even more favorable. At each time step of the optimization, the motor-side torque and speed limits are enforced using the instantaneous value of $k_{J}\left(q_{2}\right)$, so that the nonlinear effect of the varying reduction ratio is explicitly taken into account rather than approximated by a constant gain.

The vertical velocity of the CoM is

(12) $${\dot{y}}_{\mathrm{CoM}}\left(t\right)=J\;\left(q_{2}\left(t\right)\right)\;{\dot{q}}_{2}\left(t\right).$$

When the contact force vanishes, the CoM vertical acceleration equals −g and the robot enters free flight. The CoM acceleration is

(13) $${\ddot{y}}_{\mathrm{CoM}}\left(t\right)=\dot{J}\;\left(q_{2}\left(t\right)\right)\;{\dot{q}}_{2}\left(t\right)+J\;\left(q_{2}\left(t\right)\right)\;{\ddot{q}}_{2}\left(t\right).$$

**Algorithm 1** Knee joint design parameter selection via explosive jump control**Require:** A set of knee design parameters $\left\{P_{1},P_{2},\ldots ,P_{n}\right\}$**Ensure:** Maximum takeoff energy and the corresponding structure parameter  1:max_energy ← −∞  2:optimal_param ←  None  3:**for** i←1 to *n* **do**  4:    $energy\;\leftarrow \;\mathrm{simulateJump}(P_{i})$                ▹Simulate explosive jump for parameter $P_{i}$ and record takeoff energy  5:    **if** energy > max_energy **then**  6:        max_energy ← energy  7:        $optimal\_param\;\leftarrow \;P_{i}$  8:    **end if**  9:** end for** 10:**return** max_energy,optimal_param

Since no joint work can increase height once the robot is airborne, we evaluate jumping capability by the mechanical energy at liftoff, i.e., the sum of potential and kinetic energies:

(14) $$W_{\mathrm{takeoff}}=\frac{1}{2}\;m_{\mathrm{tot}}\;{\dot{y}}_{\mathrm{CoM}}^{2}\left(t_{\mathrm{to}}\right)+m_{\mathrm{tot}}\;g\;y_{\mathrm{CoM}}\left(t_{\mathrm{to}}\right)\;.$$

This energy is equivalent to the maximum reachable CoM apex height,

(15) $$H_{\max}=y_{\mathrm{CoM}}\left(t_{\mathrm{to}}\right)+\frac{{\dot{y}}_{\mathrm{CoM}}^{2}\left(t_{\mathrm{to}}\right)}{2g}=\frac{W_{\mathrm{takeoff}}}{m_{\mathrm{tot}}\;g}\;,$$

where the second equality holds when the ground reference is y=0.

Accordingly, the optimization objective is

(16) $$\max_{r,S_{0},\Delta \theta }\;W_{\mathrm{takeoff}}\;,$$

which is equivalent to

(17) $$\min_{r,S_{0},\Delta \theta }\;f\left(r,S_{0},\Delta \theta \right)=-\;W_{\mathrm{takeoff}}\;,$$

where *r*, $S_{0}$, and Δθ are the VRR knee-joint design parameters. The structural bounds are

(18) $$s.t.\;r_{\min}\leq r\leq r_{\max},\;S_{0,\min}\leq S_{0}\leq S_{0,\max},\;\Delta \theta_{\min}\leq \Delta \theta \leq \Delta \theta_{\max}.$$

### 4.2. Optimization Results and Analysis

To provide guidance for a full-scale design, the simplified model in Section 2.2 is parameterized as $l_{1}=l_{2}=0.45\;m$, $m_{1}=1.77\;\mathrm{kg}$, $m_{2}=3.16\;\mathrm{kg}$, and $m_{3}=20\;\mathrm{kg}$, with a total mass approximately half that of a full-scale humanoid robot. All links are assumed rigid with uniform mass distribution and centroids at their geometric centers. To ensure mechanical feasibility, the design parameters are restricted to r∈[25,75]mm, $S_{0}\in \left[100,250\right]\;\mathrm{mm}$, and $\Delta \theta \in [-3^{\circ },\;3^{\circ }]$. The knee actuator is a 72 V a *TQ-ILM8526SP* motor, with the characteristics shown in Figure 1.

To compare EVRR-KJ and FRR-KJ across initial takeoff angles $q_{2,0}$, we optimize the structural parameters for both and summarize the results in Table 2. The theoretical takeoff height is

(19) $$H=\frac{W_{\mathrm{takeoff}}}{m_{\mathrm{tot}}\;g}-y_{\mathrm{CoM},s}.$$

**Figure 7:**
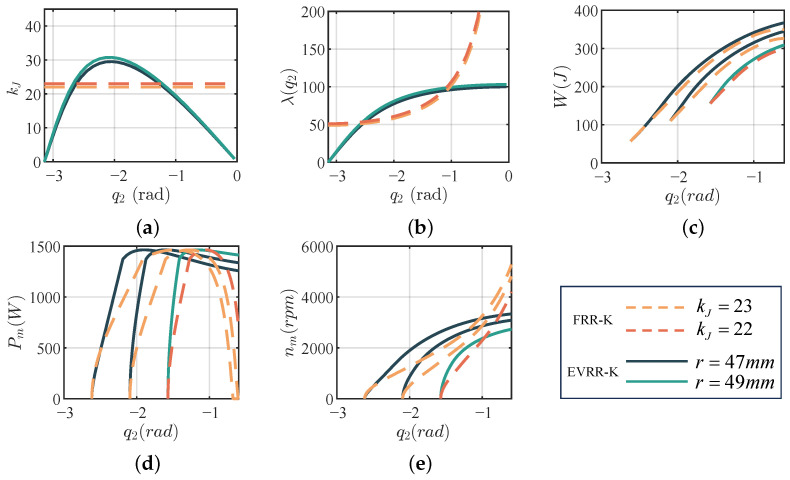
Numerical jump simulations comparing the optimized FRR-KJ and EVRR-KJ on a 1-DoF leg-like platform across various initial knee angles. (**a**–**e**) illustrate key performance metrics: joint reduction ratio $\left(\eta_{m}\right)$, knee-to-CoM transmission ratio $\left(\eta_{\mathrm{CoM}}\right)$, motor speed $\left(\omega_{m}\right)$, motor power $\left(P_{m}\right)$, and motor efficiency (η). In all subplots, curves of different colors correspond to different joint reduction-ratio parameter sets. The EVRR-KJ consistently demonstrates up to approximately 31.9% improvement in simulated jump height over the optimized FRR-KJ baseline by realizing a monotonically decreasing reduction ratio.

Across all initial angles, EVRR-KJ consistently achieves higher jump heights than FRR-KJ, with the largest improvement of approximately 31.9%. The optimal reduction ratio decreases monotonically over the working range (Figure 7a). The knee-to-CoM transmission ratio for EVRR-KJ remains nearly constant at about 100, while that of FRR-KJ increases from 50 to over 200 (Figure 7b), indicating reduced late-phase motor speed demand for EVRR-KJ. It is worth noting that the FRR-KJ baseline is optimized under the same simplified leg model, motor model, and explosive jump control strategy as EVRR-KJ, so the differences in Table 2 and Figure 7 reflect the intrinsic trade-off associated with fixed versus variable reduction ratios rather than differences in actuation or control. Taken together, Figure 7a,d,e show that the EVRR-KJ profile effectively reconciles the need for high takeoff torque with the constraint of limiting late-phase motor speed by combining a high initial reduction ratio with a progressive decrease during extension.

From Figure 7d, this qualitative behavior is reflected quantitatively: EVRR-KJ exhibits a faster early-phase power rise and a slower late-phase decay than FRR-KJ. Combining Figure 7a,e, the early high power arises from a larger reduction ratio that yields greater CoM thrust, whereas the progressive ratio decrease during extension prevents excessive motor speed and high-loss operation in the late phase. At liftoff, EVRR-KJ maintains motor speed near 3000 rpm, while FRR-KJ requires about 4000 rpm or higher, matching the design intent of angle-coupled reduction for jumping.

In addition, even with EVRR-KJ, where the reduction ratio decreases with knee extension, power still declines as joint speed increases; however, both the rate and the magnitude of this decline are markedly smaller than with a fixed-ratio joint (Figure 7d). Consequently, the high-power window is enlarged, and motor power utilization during the jump is improved.

## 5. Experiments

This section presents experimental validation of the proposed EVRR-KJ design paradigm. We conducted experiments on a one-degree-of-freedom (1-DoF) leg-like platform and on a full-scale humanoid robot to assess how the paradigm enhances knee-motor performance during jumping and improves jumping capability.

### 5.1. 1-DoF Leg-like Platform Experiments

#### 5.1.1. Platform Design and Control

**Figure 8:**
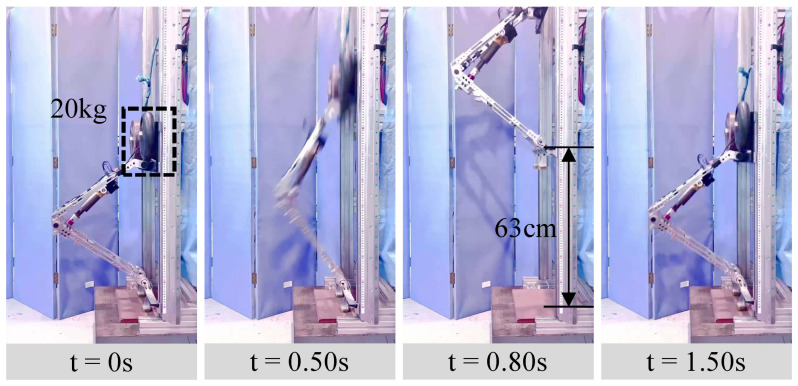
Vertical jump experiment on the 1-DoF leg-like platform, actuated solely by the primary knee joint (other joints passive). The knee joint is driven by a 72 V TQ-ILM8526SP motor; the motor-side peak power is 1.5 kW and the peak torque is 9.37 Nm.

To validate effectiveness while avoiding interference from other joints, we construct the 1-DoF platform in Figure 8 for vertical-jump experiments. The platform is driven by an EVRR-KJ joint, with all other joints being passive; its model is described in Section 2.2. System parameters are $l_{1}=l_{2}=0.42\;m$, $m_{1}=1.77\;\mathrm{kg}$, $m_{2}=3.16\;\mathrm{kg}$, and $m_{3}=20\;\mathrm{kg}$. EVRR-KJ parameters are obtained using the optimization method in Section 4 under mechanical constraints; the final values are $S_{0}=0.259\;m$ and r=0.047m. The designed reduction-ratio–knee-angle profile is shown in Figure 7a. The actuator comprises a TQ-ILM8526SP motor (TQ-Systems GmbH/TQ-RoboDrive, Seefeld, Bavaria, Germany), a 10mm lead ball screw, and an 18-bit encoder; the mechanism is illustrated in Figure 4.

The control system uses an NUC8 computer and an Elmo motor drive for real-time control at a 1 kHz loop rate. The jump controller comprises three phases: takeoff, mid-air, and landing. During takeoff, a force-controlled PD controller first brings the platform to the initial angle, after which a maximum-torque profile is applied by commanding a constant *q*-axis current $I_{q}=92\;A$ (at a bus voltage of 72 V) until the takeoff-detection angle is reached. The admissible motor torque at each speed is bounded by the manufacturer-provided torque–speed envelope, which effectively captures current, voltage, and thermal limits. During the mid-air and landing phases, a PD controller regulates torque to hold the joint at the set angle.

#### 5.1.2. Experimental Results and Analysis

**Figure 9:**
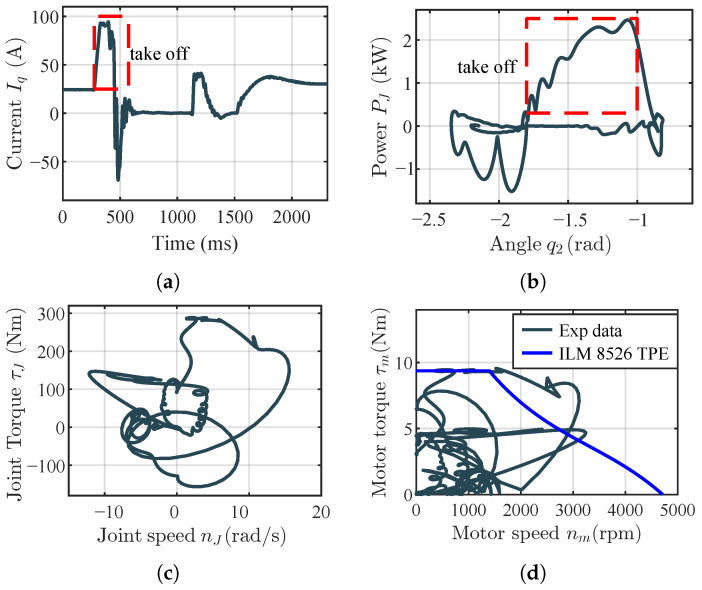
Experimental data for the explosive vertical jump on a 1-DoF leg-like platform: (**a**) actual motor current; (**b**) knee angle versus joint power; (**c**) joint torque versus joint speed; (**d**) motor torque versus motor speed. Black curves denote the measured experimental data. Blue curves represent the motor torque–speed performance envelope of the ILM 8526SP motor operating at a bus voltage of 72V. Red dashed boxes highlight the data corresponding to the takeoff phase of the jump. The results confirm that the EVRR-KJ allows the motor to deliver power well above 1.5kW over a substantial portion of the takeoff phase while keeping the operating points largely within the manufacturer-provided torque–speed envelope.

The platform has a total mass of 24.93 kg (including a 20 kg load), about half that of a full-size robot. Under these conditions, the single knee joint achieves a jump height of 0.63 m (Figure 8). To analyze motor output, the *q*-axis current $I_{q}$ is recorded by the Elmo driver, and motor position/speed are obtained from the encoder. Joint torque, angular velocity, and mechanical power are computed as $\tau_{J}=\tau_{m}k\left(q_{2}\right)$ (with $\tau_{m}=K_{T}I_{q}$), $\omega_{J}=\omega_{m}/k\left(q_{2}\right)$, and $P_{J}=P_{m,\mathrm{out}}=\tau_{J}\omega_{J}$, respectively. Results are shown in Figure 9.

Figure 9 reports the EVRR-KJ data during jumping. In Figure 9a, a large positive current is commanded at takeoff to generate high torque; after liftoff the current reverses to swing the leg; during flight the current remains near zero; upon landing, current is applied for damping. Figure 9b shows the joint power during takeoff. Power quickly exceeds 1.5kW and remains above this level through most of the late takeoff, indicating that EVRR-KJ both accelerates the power ramp-up and enlarges the high-power window. The peak reaches ∼2.5kW, exceeding the motor’s nominal rating and reflecting short-term overload capability, which also explains why the realized jump height is higher than the simulated prediction. The rapid current drop near the end of motion is consistent with momentary operation beyond the intended envelope, where current/voltage limits curtail output.

Although the peak power is high, the brief oscillations visible in Figure 9b occur only in the early phase of takeoff. As seen in Figure 9a, the commanded $I_{q}$ is smooth during this period, indicating that the power fluctuations originate from speed disturbances rather than from current noise. These speed disturbances result from uneven loading on the linear guide rail under large initial acceleration: the leg acceleration has components both upward and toward the rail, increasing the normal force and thus the tangential friction force. This induces stick–slip behavior along the rail, causing the resisting force to alternate between higher and lower levels. The resulting small speed oscillations appear as power ripples but have negligible influence on the peak torque–speed point or the overall jump performance. These structural effects are not explicitly modeled in the simplified analysis but do not alter the qualitative torque–speed behavior or the main conclusions of this study.

The speed–torque curve in Figure 9c shows a peak torque of 300Nm and a peak angular speed of 15.8rad/s. The motor torque–speed trajectory (Figure 9d) stays largely within the manufacturer-specified envelope, with a maximum speed of about 3300rpm. At takeoff, the EVRR-KJ reduction ratio is (≈13). For the same joint speed, a fixed-ratio joint (FRR-KJ) would deliver only (≈121 Nm), far below the 300Nm achieved with EVRR-KJ. Matching the EVRR-KJ peak torque with FRR-KJ would require a ratio of (≈30); maintaining the same joint speed would then drive the motor to (∼7615 rpm), which is impractical for this actuator and would push it deep into the high-loss region. The brief overload behavior observed on the 1-DoF platform is not used as a design constraint and occurs only in isolated single-jump trials with ample rest intervals; accordingly, in the full-robot experiments, the operation is deliberately restricted to within the manufacturer’s envelope. These comparisons highlight how EVRR-KJ lowers the late-phase motor-speed demand, sustains higher power during takeoff, and improves jumping performance.

For completeness, we compare the simulation results in Figure 7 with the experimental data in Section 5. In both the 1-DoF test rig and the full humanoid robot, the EVRR-KJ maintains the motor within the mid-speed constant-power region during takeoff, enlarges the high-power window, and reduces the required peak motor speed—consistent with the simulated torque–speed and power trajectories. Differences in absolute values can be attributed to real-time control delays, structural compliance, guide-rail friction, and unmodeled drivetrain and power-electronics losses. This qualitative agreement demonstrates that the proposed design paradigm is robust from simulation to hardware implementation.

Additionally, as seen in Figure 9b, the late-phase motor output power exceeds the nominal peak, and Figure 9d indicates brief operation beyond the manufacturer envelope, attributable to short-term overload capability; the control stability may degrade beyond this limit. A further contributing factor to the high peak power is the slightly delayed liftoff detection: when the joint continues to extend while ground reaction forces are still acting in the same direction as the motor output, the external load assists the motor acceleration, and part of the gravitational work is converted into additional mechanical power at the joint. Early-phase irregularities in the power rise are also influenced by rail-friction fluctuations. In the 1-DoF experiments reported here, these power peaks occur over very short durations in isolated jumps with long rest intervals, and no abnormal temperature rise or performance degradation of the actuator is observed; by contrast, all full-robot experiments in Section 5.2 are operated strictly within the manufacturer-specified torque–speed envelope. These irregularities are confined to the early phase of the motion and remain within the uncertainty bounds of torque–speed estimation based on current and encoder measurements, so they do not affect the measured peak torque, peak speed, or the overall characterization of the actuator operating region.

Quantitative differences between the simulated and measured values can be attributed to real-time control delays, structural compliance, guide-rail friction, drivetrain losses, and power-electronics losses, none of which are explicitly modeled in the simplified 1-DoF analysis but all of which are present in hardware.

### 5.2. Full-Scale Humanoid Robot Jumping Experiment

**Figure 10:**
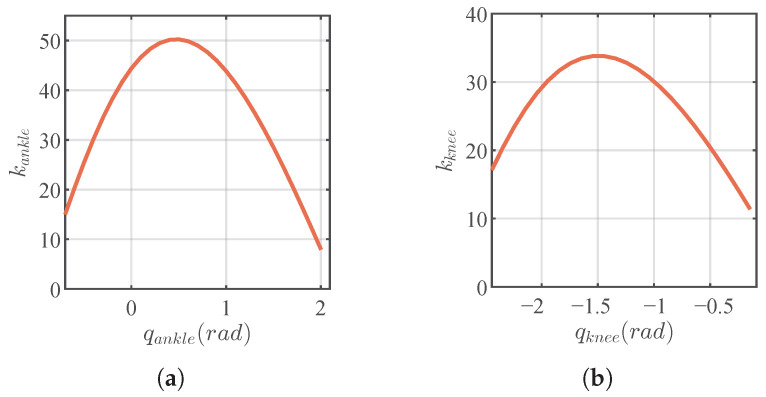
Knee and ankle joint reduction ratios of the full-scale humanoid robot. (**a**) Ankle joint reduction ratio. (**b**) Knee joint reduction ratio.

**Figure 11:**
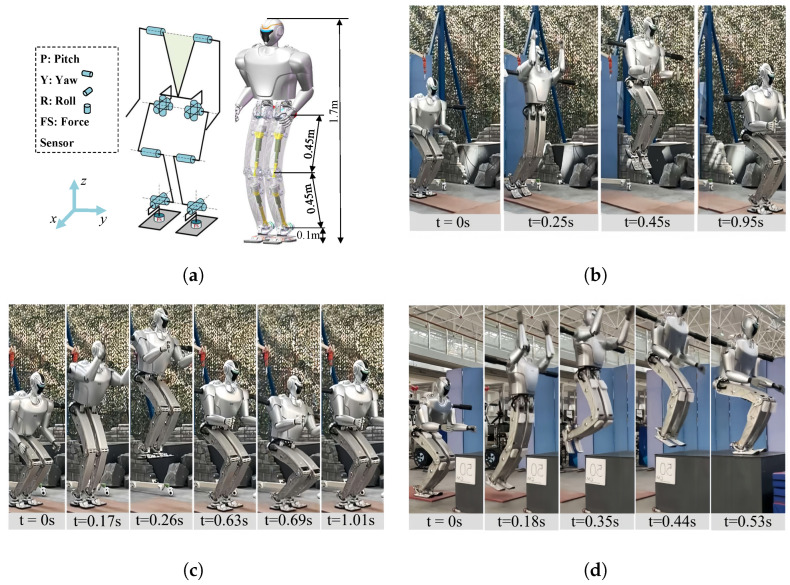
Full-scale humanoid robot (BHR8-J1) with the proposed EVRR-KJ and representative jumping experiments: (**a**) DoF configuration and dimensions. (**b**) High jump (0.5 m). (**c**) Long jump (1.1 m). (**d**) Box jump (0.5 m).

EVRR-KJ was further validated on a full-scale humanoid robot equipped with the proposed knee joint. The knee and ankle reduction ratios of the robot are provided in Figure 10. The robot configuration and representative jumping experiments are shown in Figure 11a. The robot is configured with 14 degrees of freedom (DoF): three at each hip, one at each knee, and two at each ankle (see Table 3). The total mass is 45kg. For control, an NUC8 computer and 14 Elmo drivers were used over EtherCAT with a 1ms cycle time. Each motor was instrumented with an 18-bit encoder, and the bus voltage was 72V.

Jump experiments were conducted as shown in Figure 11. For the high jump (Figure 11b), a double-stance countermovement with vertical takeoff and two-foot landing near the takeoff location was used; the metric was the maximum foot–ground clearance. For the long jump (Figure 11c), a stationary double stance with forward takeoff and two-foot landing ahead was used; the metric was the horizontal distance from toe at takeoff to heel at landing. For the box jump (Figure 11d), a double stance with two-foot landing on the top surface was used; the metric was the box height. Using the EVRR-KJ, BHR8-J1 achieved a high jump of 0.5m, a long jump of 1.1m, and a box jump onto a 0.5m platform.

**Figure 12:**
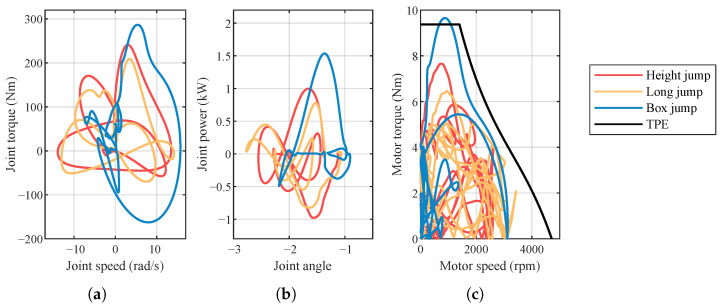
Knee joint data for BHR8-J1 during the 0.5m high jump, 1.1m long jump, and 0.5m box jump: (**a**) joint angular velocity versus joint torque; (**b**) joint angle versus joint power; (**c**) motor speed versus motor torque. In particular, the box-jump trials show that the EVRR-KJ can simultaneously achieve joint torques up to approximately 286Nm and joint speeds around 148rpm, while keeping the motor speed below approximately 3000rpm, which would be difficult to realize with a single fixed reduction ratio.

To further assess the contribution of EVRR-KJ to jumping, knee-joint data were collected and analyzed over full-jump trials; the acquisition and processing followed the procedure in Section 5.2. Three tasks were evaluated: a 0.5m high jump, a 1.1m long jump, and a 0.5m box jump. Motor torque, motor speed, joint torque, joint speed, and mechanical power were obtained as shown in Figure 12. Because the left–right results were similar, the right-leg data are reported.

From Figure 12, the box jump imposes the highest knee demands among the three tasks: the peak joint torque reaches 286Nm, the peak joint speed reaches 148rpm, and the peak mechanical power reaches 1.5kW. These values are near the limits of the TQ-ILM8526SP actuator (motor-side peak torque 9.37Nm, nominal peak power ≈1.5kW). While short-term overload capability is evident in the single-joint platform tests, loss of control is also observed beyond that range; accordingly, the full-robot trials are operated within the manufacturer’s envelope.

A fixed-ratio knee (FRR-KJ) was estimated to be unable to meet these concurrent torque–speed demands. Matching the 286Nm peak torque would require a reduction ratio of (≈30.5). At this ratio, achieving a 148rpm knee speed would drive the motor to (∼4500 rpm), i.e., near the no-load speed, pushing operation into a high-loss region and causing a rapid power drop late in takeoff. By contrast, as illustrated in Figure 12c, EVRR-KJ maintains motor speed below 3000rpm. The large early-phase ratio provides sufficient acceleration, whereas the smaller late-phase ratio prevents entry into the high-loss region and avoids excessive torque roll-off. These results substantiate the advantage of EVRR-KJ in sustaining high power during takeoff and improving jump performance.

From the 0.5m box-jump trials, BHR8-J1 exhibited headroom for both high and long jumps: foot–ground clearance exceeded 0.6m, and horizontal landing offset exceeded 1.1m. Joint demands for the high and long jumps were lower than for the 0.5m box jump. Sustained high-power output was not observed, primarily because the robot was not operated at maximum power; the current-control and trajectory-optimization framework [10] presently does not account for dynamic joint-output limits. Future work will exploit the variable reduction ratio while explicitly modeling these limits to further enhance jumping performance.

## 6. Conclusions and Future Work

This paper proposed a variable-reduction-ratio knee-joint paradigm that couples the reduction ratio to the joint angle to enhance power delivery and jumping performance. By aligning the transmission with the knee kinematics, the approach enables high power over a larger portion of the jump phase, mitigating the limitations observed with the optimal fixed-ratio baseline used in this study. A linear-actuator-driven guide-rod mechanism was developed to implement the strategy, and an optimization framework guided by explosive jump control was introduced. Experimental validation showed a vertical jump of 0.63 m on a single-joint test platform, representing an approximately 31.9% improvement over the optimal fixed-ratio design under the tested conditions. Integration into a full-scale humanoid robot enabled a long jump of 1.1 m and a high jump of 0.5 m, confirming the effectiveness and practical applicability of the methodology.

The present study did not address the role of the ankle in jump dynamics. Future work will investigate ankle actuation and control (e.g., variable reduction, compliance, and coordinated knee–ankle control) to further improve explosive jumping performance and agility. In addition, future work will include implementing and testing alternative knee transmission schemes on the same platform as additional hardware baselines, conducting more systematic sensitivity analyses with respect to model parameters and drivetrain losses, and deepening the biomimetic analysis by more directly relating the EVRR-KJ design to human knee moment-arm and joint-power profiles during jumping. More broadly, the present experimental validation is limited to a single actuator configuration and a representative set of jumps, and does not include detailed statistical analysis, long-term thermal modeling, or durability testing; expanded dynamic testing, the explicit modeling of efficiency variation and heat dissipation, and the systematic assessment of actuator wear will therefore also be important topics for future work.

## Figures and Tables

**Table 2:** Knee joint parameter optimization results for explosive jump control on a 1-DoF leg-like platform.

Joint Type	Initial Angle (Rad)	Optimal Parameters $(r,S_{0},\Delta \theta )/k$	Jump Height ^a^ H(m)
	−2.6180	(47,150,0)	0.62
EVRR-KJ	−2.2689	(47,150,0)	0.51
	−1.9199	(49,150,0)	0.37
	−2.6180	22	0.47
FRR-KJ	−2.2689	22	0.40
	−1.9199	23	0.34

^a^ The jump height *H* from simulation is the maximum vertical CoM distance from the ground.

**Table 3:** Actuation configurations of the BHR8-J1 joints.

Joint (Axis)	Actuator Type	Ratio/Lead	Motor Model
Hip (Yaw)	Harmonic drive	100:1	TQ-ILM5014SP
Hip (Roll)	Harmonic drive	100:1	TQ-ILM8513SP
Hip (Pitch)	Planetary gearbox	24.695:1	TQ-ILM8523SP
Knee (Pitch)	Ball screw	10 mm lead	TQ-ILM8526SP
Ankle (Pitch)	Ball screw	5 mm lead	TQ-ILM7018SP
Ankle (Roll)	Harmonic drive	50:1	TQ-ILM5014SP

## Data Availability

Data are contained within the article.

## Abbreviations

The following abbreviations are used in this manuscript:

EVRR-KJ	Explosive Variable Reduction Ratio Knee Joint
FRR-KJ	Fixed Reduction Ratio Knee Joint
TPE	Torque Performance Envelope
PPE	Power Performance Envelope
